# Host Immune Responses and Immune Evasion Strategies in African Trypanosomiasis

**DOI:** 10.3389/fimmu.2019.02738

**Published:** 2019-11-22

**Authors:** Chukwunonso Onyilagha, Jude Ezeh Uzonna

**Affiliations:** ^1^Department of Immunology, Rady Faculty of Health Sciences, Max Rady College of Medicine, University of Manitoba, Winnipeg, MB, Canada; ^2^Department of Medical Microbiology, Max Rady College of Medicine, Rady Faculty of Health Sciences, University of Manitoba, Winnipeg, MB, Canada

**Keywords:** African trypanosomes, immunity, immunosuppression, immune evasion, resistance, susceptibility

## Abstract

Parasites, including African trypanosomes, utilize several immune evasion strategies to ensure their survival and completion of their life cycles within their hosts. The defense factors activated by the host to resolve inflammation and restore homeostasis during active infection could be exploited and/or manipulated by the parasites in an attempt to ensure their survival and propagation. This often results in the parasites evading the host immune responses as well as the host sustaining some self-inflicted collateral tissue damage. During infection with African trypanosomes, both effector and suppressor cells are activated and the balance between these opposing arms of immunity determines susceptibility or resistance of infected host to the parasites. Immune evasion by the parasites could be directly related to parasite factors, (e.g., antigenic variation), or indirectly through the induction of suppressor cells following infection. Several cell types, including suppressive macrophages, myeloid-derived suppressor cells (MDSCs), and regulatory T cells have been shown to contribute to immunosuppression in African trypanosomiasis. In this review, we discuss the key factors that contribute to immunity and immunosuppression during *T. congolense* infection, and how these factors could aid immune evasion by African trypanosomes. Understanding the regulatory mechanisms that influence resistance and/or susceptibility during African trypanosomiasis could be beneficial in designing effective vaccination and therapeutic strategies against the disease.

## Introduction

African trypanosomiasis is a disease caused by extracellular hemoprotozoan parasites that belong to the genus *Trypanosoma*. Trypanosomes are unicellular parasites that are equipped with flagella which help with their movement (1). The disease is associated with serious health and economic problems in the affected countries, and can be fatal if not properly treated (2, 3). The human form of the disease is caused by *Trypanosoma brucei rhodesiense* and *Trypanosoma brucei gambiense* while the animal form of the disease is mostly caused by *Trypanosoma congolense, Trypanosoma vivax*, and *Trypanosoma brucei brucei*. Trypanosomiasis is a vector borne disease. The transmission of the parasites from one host to another occurs during a blood meal by several species of tsetse flies belonging to the genus *Glossina*. For both human and animal African trypanosomiasis, *Glossina morsitans, Glossina tachinoides, Glossina fuscipes, Glossina pallidipes, Glossina swynnertoni*, and *Glossina palpalis* are some of the important vectors responsible for transmission due to their wide distribution in countries where the disease is endemic (4), and their presence is often used as a key predictor of the disease.

Although it has been estimated that 65 million people living in 36 countries in sub-Saharan Africa are at risk of contracting the disease, the number of reported cases per year dramatically reduced (~10,000 new cases annually) in 2009 due to increasing efforts to combat the disease (5–7). In 2018, the number of reported cases further reduced to 977, an almost 90% decline in 10 years (8). However, the real number of cases may be grossly underestimated because the disease is mostly found in rural communities in the endemic areas, and it has been estimated that only about 10% of the affected people living in these areas are accounted for (9, 10). In other words, the majority of the cases remain either undiagnosed or unreported, suggesting that the disease impact and statistics could be worse than currently believed.

Human African trypanosomiasis affects both the young and old especially those that engage in farm-related activities in the endemic rural areas, although few reported cases have also been reported in urban areas (11). This is most likely related to favorable environmental conditions in rural areas that favor breeding of the insect vector. Woody vegetations are known to support tsetse fly abundance as the flies tend to rest on tree trunks during the hot humid day. Efforts toward eliminating the disease were almost successful in the 1960s, but because of political instabilities and accompanying poor surveillance, the eradication process was disrupted, which allowed the disease to re-emerge (12–15). Economically, the threat posed by African trypanosomiasis to animals is much more than that posed to humans (16). The disease has been linked to severe food and economic loss in the affected regions (2, 3).

## Routes of Infection in Experimental African Trypanosomiasis

Natural infection with African trypanosomes starts with intradermal injection of parasites along with tsetse saliva into the mammalian host by an infected tsetse fly. Following the introduction of the parasite into the dermis, the parasites undergo several transformations at this site with associated inflammatory response resulting in the development of chancre (1). These events precede the entry of the parasites into the blood stream of the infected host. Human African Trypanosomiasis usually develops from hemolymphatic phase to meningo-encephalitic phase; the meningo-encephalitic stage is one of the hallmarks of the disease and is often associated with severe alteration in the sleep-wake cycle (17). Cerebral infections with African trypanosomes in animals have also been documented; while *T. congolense* and *T. vivax* rarely invade the central nervous system (CNS), *T. brucei brucei* has been recovered from cerebrospinal fluid (CSF) and are able to cause central nervous system (CNS) impairment (18). Although there has been a report of *T. congolense* being found in the CSF, this was speculated to be aided by a mixed infection with other species (19). The tsetse fly saliva has been proposed to enable the blood-feeding process, promotes parasite transmission, and possesses powerful immunomodulatory properties including skewing T helper cell responses and anti-/proinflammatory properties (20–23). In contrast to this, the overwhelming majority of experimental infection studies utilize the intraperitoneal infection route, which involves inoculation of blood stream forms directly into the peritoneal cavity. It is conceivable that the use of intradermal route in experimental African trypanosome infection would capture some of the series of early events that occur during a natural infection (24). Therefore, it is likely that the intraperitoneal route of infection (as is used during most experimental infections) may not clearly represent the early immune response that occurs during natural infection when the vector bites their human or animal hosts (24). Indeed, it has been shown that intradermal infection of experimental animals is able to induce the activation of some immune mediators that are distinct from those seen during intraperitoneal infection (24, 25). Therefore, the use both the intradermal and intraperitoneal infection routes of infection (when possible) in experimental African trypanosomiasis would be helpful in comparing the immune responses due to different infection routes. This would enable us to further identify the missing links that could help in the understanding of the host-parasite interactions that regulate disease outcome.

## Key Components of Immune Response to African Trypanosomes

Both the innate and adaptive components of the immune system play crucial roles in resistance to African trypanosomiasis. Although African trypanosomes are free living in the bloodstreams of their mammalian host, and are therefore direct targets of antibody-mediated destruction, experimental animal models of infection show that a full component of the immune system (innate and adaptive) are critical for the development of optimal resistance to the infection.

### Macrophages

Macrophages are one of the most important cells that contribute to innate immunity to African trypanosomiasis. They are capable of influencing the adaptive immune response directly through antigen presentation or indirectly by secreting many effector molecules including cytokines. During infection with African trypanosomes, classically-activated macrophages have been shown to contribute to parasite clearance via the process of phagocytosis (26), as well as through the production of proinflammatory cytokines and nitric oxide (27–31). Macaskill et al. conducted a study to specifically examine the roles played by antibody, macrophage activation, and complement in parasite clearance using trypanosomes labeled with [75Se]-methionine. Their results showed that clearance of parasites from circulation was mostly dependent on antibody-mediated phagocytosis by liver macrophages (32).

Both classical and alternative macrophage activation occur during African trypanosomiasis, and their effects vary depending on the timing of their activations. Enhanced survival of trypanosome-infected mice has been associated with the ability to switch from classically-activated macrophages (M1) at the early stages of infection to an alternatively-activated phenotype (M2) during the advanced stages of infection (33, 34). This switch from M1 to M2 macrophages is essential because if left unchecked, classically-activated macrophages produce excessive amounts of proinflammatory cytokines that induce immune hyperactivation, causing collateral tissue damage and death in trypanosome-infected animals (35, 36). Thus, the inability of mice to upregulate alternative macrophage activation while concomitantly downregulating classical activation during the advanced stage of *T. congolense* infection led to enhanced susceptibility to infection, which was associated with excessive production of proinflammatory cytokines and early death of infected mice (37).

Several studies have associated pathology—mostly anemia and cachexia—during African trypanosome infection with the over-activation of macrophages and the resulting release of harmful molecules such as TNF-α and nitric oxide (38, 39). However, the underlying mechanisms involved in this process are still not fully investigated. While TNF-α is directly linked to the development of anemia in *T. brucei* infection, it appears not to be involved in *T. congolense* infection. Instead a direct alteration of red blood cells has been linked. (38–42).

The roles of macrophages during intradermal infection have not been well-investigated. Wei et al. demonstrated that mice that are deficient in inducible nitric oxide synthase (iNOS) were susceptible to intradermal low dose *T. congolense* infection (24), and proposed that macrophages are at the center of innate control of primary intradermal infection. However, results from our lab have shown that depletion of macrophages before *T. congolense* infection did not alter the resistance of mice to primary intradermal infection (25). This observation suggests that host factors other than macrophages are responsible for mediating early resistance observed during intradermal infection. More studies are needed to fully determine the roles played by macrophages in enhanced resistance observed during intradermal infection.

### Complement

Although African trypanosomes have different mechanisms to avoid lysis by complement, the activation of this important arm of the innate immune defense system is considered to be critical for mediating parasite clearance in infected animals. Both the classical and alternative pathways of complement system are activated during infection with African trypanosomes. Activation of the classical pathway is mediated by specific antibodies against the parasite and has been reported to contribute to parasite clearance via antibody-mediated lysis, opsonization and phagocytosis (43). The antibody-independent alternative pathway is usually activated during the early stages of infection when specific antibodies have not been formed, and has also been shown to contribute to parasite clearance (44).

Although phagocytosis of African trypanosomes by macrophages can occur *in vitro* in the absence of complement (45), the efficiency of parasite clearance and immune complex removal is greatly enhanced in the presence of complement (46). Indeed, it was demonstrated that depletion of the complement component C3 by treatment with cobra venom factor leads to significant reduction in hepatic uptake of opsonized trypanosomes (32). However, other studies showed that partial depletion of C3 did not affect either parasitemia control of *T. brucei brucei* or phagocytosis of *T. brucei rhodesiense* (47). Jarvinen and Dalmasso found that parasite control could not be attributed to the presence of C3, C5, or late acting complement factors (48). In addition, Jones and Hancock found no marked differences in the survival periods of C5-deficient and C5-sufficient mice infected with African trypanosomes (49). Collectively, these findings suggest that complement activation may be required but not critical for resistance to African trypanosomes. However, given that the cleavage products of C3 and C5 (C3a and C5a) have been reported to help in the initiation of inflammatory responses during infection (50), and that alternative and classical complement activation is higher in the relatively resistant C57BL/6 mice following *T. congolense* infection (51, 52), it is conceivable that complement activation may contribute to optimal resistance to certain species of African trypanosomes.

### T Cells

Both CD4^+^ (helper) and CD8^+^ (cytotoxic) T cells play a critical role in regulating the outcome of infection with African trypanosomes. CD4^+^ T cells contribute to resistance by producing cytokines that regulate other innate and adaptive immune cells, and by providing help to B cells to ensure efficient isotype class-switching and production of specific antibody responses to parasite antigens. CD4^+^ T helper cells provide signals that regulate B cell survival and differentiation into antibody producing cells (53). In support of this, Shi et al. showed that anti-parasite IgG2a production, as well as IFN-γ and IL-10 levels, was impaired in *T. congolense*-infected CD4^+^ T cell deficient mice (54). Following this observation, Tabel et al. proposed that future vaccines against African trypanosomiasis should be targeted toward encouraging the generation of T helper 1 cells (Th1) that would support B cells in class-switching from IgM to IgG2a during infection (55). Interestingly, it was found that CD4^+^ T cell deficient mice infected with *T. congolense* had significantly lower parasitemia and prolonged survival period compared with their WT mice, suggesting that CD4^+^ T cells might also contribute to disease pathogenesis and death in infected mice (54).

The role of CD8^+^ T cells has been controversial in African trypanosomiasis. Earlier studies suggested that polyclonal activation of CD8^+^ T cells by *T. brucei brucei*-derived T lymphocyte triggering factor (TLTF) leads to massive release of IFN-γ, which is responsible for profound immunosuppression and susceptibility to the infection (56, 57). However, a study by Wei and Tabel showed that the beneficial effect of anti-CD25 treatment in mice during *T. congolense* infection was lost upon depletion of CD8^+^ T cells (58), suggesting that CD8^+^ T cells may be playing a protective role during *T. congolense* infection. In another study that investigated the relative contributions of CD4^+^ and CD8^+^ T cells in *T. brucei* infection, Liu et al. (59) reported that IgG antibody synthesis was dependent on CD4^+^ T cells and not CD8^+^ T cells. In addition, they showed that infected CD8^+^ T cell-deficient mice had lower parasitemia and survived significantly longer than their WT counterpart mice. However, this enhanced survival was lost upon depletion of CD4^+^ T cells. Furthermore, cytokine (IFN-γ and IL-10) production during infection was also attributed to CD4^+^ but not CD8^+^ T cells (59). These observations indicate that CD8^+^ T cells mediate susceptibility while CD4^+^ T cells mediate protection during infection with *T. brucei brucei*.

Our understanding of the roles played by T cells has been simplified by the Th1 (proinflammatory properties) and Th2 (anti-inflammatory properties) paradigm. Overall, the control of parasites during infection with African trypanosomes is believed to be associated with a Th1 response during the early phase of infection and a switch to a Th2 phenotype in the advanced stage of infection (33, 34). In support this, *T. congolense*-infected TSLPR^−/−^ mice with impaired Th2 response are highly susceptible to *T. congolense* infection during the chronic stage of the disease, and died significantly earlier than their WT controls (37). This susceptibility was associated with increased production of proinflammatory cytokines by CD4^+^ T cells from these mice, which was reversed upon treatment of the infected TSLPR^−/−^ mice with anti-IFN-γ monoclonal antibody (37).

### B Cells

Because African trypanosomes are extracellular bloodstream parasites, they are constantly exposed to humoral immune factors and are direct target for antibody-mediated destruction. Indeed, B cell deficient mice are highly susceptible to African trypanosomes (60). In addition, passive transfer of variant-specific antibodies or B cells (but not T cells) to immunocompromised mice results in variant-specific protection (60, 61). Furthermore, the differential resistance observed in several strains of mice and cattle following infection with African trypanosomes has been attributed to differences in the production of parasite-specific antibodies (62–64). A study using mice that lack Bam32 further demonstrated the importance of strong B cell response for protection against infection with African trypanosomes (65). Bam32 is a B cell adaptor protein that plays a critical role in B cell activation (66), survival (67), and antigen presentation (68). Bam32 deficient mice on a relatively resistant background were more susceptible than their WT counterpart mice to *T. congolense* infection and showed impaired germinal center response as well as significantly low levels of parasite-specific IgG antibodies in the serum (65, 68).

B cells become activated upon encountering their cognate antigens, and this is followed by the initiation of germinal center formation with the help of follicular CD4^+^ T helper cells (Tfh) (69). Germinal centers are large areas in the secondary lymphoid organs where intense B activities such as proliferation, somatic hypermutation, selection, and class switch recombination take place, resulting in the production of various antibody isotypes with high antigen binding affinity (53). The requirement of B cells during infection with African trypanosomiasis centers on optimal activation, efficient germinal center (GC) formation, and production of strain-specific antibodies.

One of the hallmarks of African trypanosomiasis is excessive polyclonal activation of B cells leading to increased serum levels of trypanosome-specific and non-specific antibodies, including heterophilic and autoantibodies (70, 71). These observations led to the postulation that African trypanosomes possess molecules (VSG for instance) that could non-specifically activate B cells to produce antibodies (70). During infection with African trypanosomes, both T cell-dependent and T cell-independent antibody response are produced against the variant and invariant VSG molecules, cytoplasmic, and other nuclear parasite antigens (72). However, the overall quality (as assessed by binding affinity, isotype, and quantity) of the response is increased in the presence of T cells (72). For example, although IgM anti-VSG antibodies (which are mostly produced in a T cell-independent manner) can mediate parasite clearance, the different subclasses of IgG antibodies, whose production are T cell dependent, mediate a more effective parasite clearance in both mice and cattle compared to IgM (62, 73).

### Cytokines

The profile and magnitude of cytokines produced during African trypanosomiasis play a critical role in determining susceptibility and resistance to the disease. Although the contributions of some cytokines in the pathogenesis of African trypanosomiasis have been demonstrated in different experimental settings, it is challenging to fully determine the precise role of specific cytokines in disease pathogenesis because of their pleiotropic activities (55). Studies have shown that infection with African trypanosomes leads to massive production of proinflammatory cytokines in the infected mammalian host. The initial inflammatory response during infection usually leads to the release of proinflammatory mediators like TNF-α, IL-1, IL-6, and NO by classically activated macrophages, and these have been shown to play important roles in mediating early protection during infection (26, 30, 74–78). Other cytokines like IL-12, MCP-1, IL-10, IFN-γ, and IL-4 have also been shown to mediate either pro-inflammatory or anti-inflammatory activities during infection (26, 54, 79–83).

Although the initial outburst of inflammatory cytokines is essential for resistance, it requires regulation to prevent collateral tissue damage. The switch from classically activated to alternatively activated macrophages later during infection by type II cytokines such as IL-4, IL13, and TSLP is critical for maintaining a Th2 type environment which have anti-inflammatory properties (1, 37). In fact, protection during infection with African trypanosomes is associated with the ability to switch from an early Th1 to Th2 response during the later stages of infection (33, 34, 37, 84). In line with this, the absence of TSLP signaling (which is a key cytokine that drives Th2 differentiation), in *T. congolense*-infected mice led to the inability to control more than two waves of parasitemia and early death compared to their wild-type counterpart mice (37). This inability to effectively control parasitemia was associated with overproduction of proinflammatory cytokines (including IFN-γ and TNF-α) and impaired activation of alternatively activated macrophages (37).

The effect of cytokines in the pathogenesis of African trypanosomiasis is complex and depends on the quantity and time of production during the infection (55). For example, while the production of IFN-γ and TNF-α are critical for protection during infection with African trypanosomes (85–88), their production in excessive amounts is detrimental and leads to susceptibility and death of infected mice (27, 79, 82). For instance, while IFN-γ-deficient mice are highly susceptible to *T. brucei* or *T. congolense* infection and fail to control the first wave of parasitemia (85, 87), neutralization of IFN-γ by antibody treatment during *T. congolense* infection is associated with enhanced resistance (lower parasitemia and prolonged survival) of the highly susceptible mice (79). Furthermore, acute death of infected relatively resistant mice following treatment with anti-IL-10 receptor antibody was completely abrogated by co-treatment with anti-IFN-γ mAb (82). These observations suggest that IFN-γ is a key mediator of death in these mice.

Another key cytokine that regulates the outcome of infection with African trypanosomes is IL-10. Due to its anti-inflammatory properties, IL-10 acts to downregulate excessive effector activities of both T cells and macrophages (82, 89), which are key cells that are involved in the production of inflammatory cytokines following infection with African trypanosomes. In *T. congolense*-infected cattle, reduced nitric oxide production and increased IL-10 and IL-4 mRNA levels were linked to protection (73, 90).

## Immune Evasion Strategies used by African Trypanosomes

### Antigenic Variation

Adaptation mechanisms within the host are known to exist among bacteria, parasites, and viruses. Antigenic variation is one of the hallmarks of African trypanosomes and constitutes a major adaptation mechanism of evading their host immune response. In fact, it has been suggested that the inability to fully understand the mechanisms that regulate antigenic variation during infection with African trypanosomes is one of the major obstacles standing in the way of developing a vaccine for African trypanosomiasis (38).

Upon injection into the skin, the parasites grow and multiply in the bloodstream of their mammalian host. Because African trypanosomes are completely extracellular in nature, they are continuously exposed to the host's humoral immune defenses. Although clone-specific antibodies are effective in mediating parasite clearance (62, 85), the natural ability of the parasites to undergo antigenic variation during the course of infection renders the antibodies ineffective at mediating cure. The expression of new VSG allows the parasites to evade antibody-mediated destruction thus permitting them to grow and multiply, and requiring the host to initiate new antibody responses against the emerging new clones.

Bloodstream forms of African trypanosomes are covered with a densely packed protective coat comprising of over 10^7^ copies of variant surface glycoprotein (VSG) molecules (1). These millions of identical glycoprotein molecules function to prevent complement-mediated lysis of the parasites (91, 92). They are attached to the surface of the plasma membrane of the parasite via the glycosylphosphatidylinositol (GPI) anchor (93). Although mostly membrane bound, soluble forms of VSG are released into the circulation upon cleavage of the GPI anchor by parasite-encoded phospholipase C (PLC) known as GPI-PLC (94). This process has been reported to trigger some inflammatory responses during infection (95).

Trypanosomes contain hundreds of VSG genes (constituting about 10% of the entire parasite genome) with only about 7% being fully functional (96). The transcription of VSG genes occurs at a telomere of the chromosomes containing the VSG expression sites (97). Because only one expression site can be active at any given time, only one of the VSG molecules is expressed on the surface coat of the parasite leading to identical display of surface coats (1). In addition, because antibody response is made only to this particular antigenic type that is being expressed, a switch in VSG expression would lead to the initiation of new antibody response, a condition that could subsequently pave the way for immune exhaustion due to the continuous need to mount immune response to numerous VSG-expressing clones. Trypanosomes are able to control VSG gene expression by turning off an active expression site (and turning on a previously silent expression site) and by rearrangement of the VSG genes mostly by reciprocal recombination and gene conversion (98–100). Efforts targeted at disrupting the VSG switching by the parasites could be a major step in designing effective disease control strategies.

### Polyclonal B Cell Activation

Because of their extracellular life style, B cells play a critical role in clearance of African trypanosomes from the blood of infected hosts by producing parasite-specific antibodies. As a result, they have developed mechanisms to suppress and evade the host specific antibody responses. Trypanosomes are able to exploit the ability of B cells to produce antibodies and use this to induce excessive activation of antibody-producing cells. This often leads to an increase in plasma levels of specific and non-specific immunoglobulins in infected animals. This hypergammaglobulinemia, which results from extensive B cell expansion, was first reported as a major feature of infection with African trypanosomes (101). While specific antibodies against parasite antigens are produced in both T cell-dependent and independent pathways (72), the induction of non-specific (polyclonal) B cell activation in a T cell-independent manner represents a strategy through which the parasites regulate specific antibody responses that contributes to immune evasion (102). Although not clearly known, it has also been reported that experimental *T. congolense* infection causes B cell depletion in the bone marrow as well as the periphery, which in turn impact negatively on the specificity of anti-VSG immunoglobulins produced during infection (103). The resulting impaired B cell development and maturation makes it impossible for the optimal development of memory B cell subsets that are key when considering vaccination strategies (104).

Several trypanosomal moieties, including DNA and VSG have been shown to contribute to polyclonal B cell activation following infection with African trypanosomes. Trypanosomal DNAs are able to initiate TLR-9 signaling leading to non-specific B cell activation and production of poly-specific antibodies to VSG (102, 105). Indeed, autoantibodies against red blood cells, cardiolipids, nucleic acids, rheumatoid factors, and component of CNS myelin (the galactocerebrosides) have been detected in sera of infected animals (106–111). Furthermore, the overproduction of antibodies could result in over-engagement of the Fcγ receptors on phagocytes, which would eventually contribute to impaired opsonization and phagocytosis of parasites (102, 105). In addition, an increased in a sub-population of CD5 expressing B cells have also been documented, and these cells are believed to be responsible for the excessive production of immunoglobulins and autoantibodies during *T. congolense* infection in cattle (105).

### Hypocomplementemia

African trypanosomes are able to take advantage of complement activation to evade the host immune response. Because the classical complement activation is critical for lysing parasites coated with VSG-specific antibodies, trypanosomes, in an effort to enhance their survival, are able to shed enormous VSG in the circulation which leads to the formation of immune complexes with antibodies. This “decoy mechanism” of shedding VSG prevents the deposition of membrane attack complex on the parasite cell membrane, thereby preventing complement-mediated lysis of the parasite. The continuous activation of the complement system often leads to a state of hypocomplementemia, which is a hallmark of infection with African trypanosomiasis (112). Also, due to their spatial and dense arrangement, the VSG is known to mask the binding of complement proteins on the plasma membrane leading to the inability to trigger alternative complement activation. This results in halting of the alternative pathway activation at the C3 convertase stage thereby preventing further activation of C5–C9 stages and subsequent formation of the membrane attack complex that usually initiate lysis of trypanosomes (113, 114).

### Induction of Regulatory Cells

One of the hallmarks of African trypanosomiasis is suppression of lymphocyte proliferation, and this has been reported to be one of the key factors that prevent parasite control in infected animals (115–117). Several cell types have been implicated in the pathogenesis of immunosuppression in African trypanosomiasis. Although suppressor macrophages and suppressor T cells were the earliest identified culprits (118–120), recent studies have clearly revealed additional roles played by regulatory T cells (25, 58, 121) and myeloid-derived suppressor cells (122). Schleifer and Mansfield (123) showed that macrophages suppress T cell responses through the production of reactive nitrogen intermediates and prostaglandins. Similarly, independent studies (using anti-CD25 mAb treatment) have shown that CD4^+^CD25^+^Foxp3^+^ T cells (Tregs) play a role in suppression of immune response to *T. congolense* (25, 58, 121). In these studies, immunity to the parasites (including control of parasitemia and survival) was enhanced in the absence of Tregs. Indeed, it was found that during intradermal infection with *T. congolense*, the parasites induces the expansion of regulatory T cells as a mechanism to induce immune suppression in order to evade host's immune response and enhance their survival (25).

Suppressor macrophages have also been shown to mediate suppression of host immune response as a means to enhance parasite survival. For example, trypanosomes are able to take advantage of the alternative macrophage activation to enhance their survival in the host. These parasites have been shown to preferentially induce alternative macrophage activation with the sole aim of upregulating host arginase, which has been shown to reduce the synthesis of trypanocidal nitrosylated compounds as well as upregulate L-ornithine production, a critical process in the synthesis of polyamines that is required for parasite growth (124, 125). Recently, we showed that *T. congolense* infection was associated with the induction of myeloid-derived suppressor cells, which suppressed T cell responses (proliferation and IFN-γ production) during *T. congolense* infection in an arginase-1-dependent mechanism (122). This suggests that targeting the arginase pathway during infection could limit parasite survival by increasing various effector responses directed against the parasites.

### Trypanosome Lytic Factors (TLFs)

Two species of African trypanosomes, *Trypanosoma brucei gambiense* and *Trypanosoma brucei rhodesiense*, are able to infect their human host. This is due to the parasites' ability to manipulate and evade the effect of TLFs that are known to have anti-parasite activity. TLF comprises of two serum complexes, Trypanosome Lytic Factor 1 and 2 (TLF1 and TLF2) (126, 127). These complexes are made up of primate-specific protein haptoglobin-protein (HPR) and apolipoprotein L1 (APOL1) (128). It is known that HPR is responsible for targeting TLF1 to the parasites, while APOL1 is the trypanolytic toxin (129–131). In *Trypanosoma brucei rhodesiense*, resistance to APOL1 is related to the expression of an expression site-associated gene (ESAG) generally referred to as serum resistance-associated (SRA) gene (132, 133). SRA is able to bind to APOL1, and the resulting interaction leads to the inactivation of APOL1 via the inhibition of its membrane insertion, resulting in degradation by proteases (128, 134). In *Trypanosoma brucei gambiense*, resistance is conferred through *T. b. gambiense*-specific glycoprotein (TgsGP) (135). The deletion of the gene that encodes TgsGP renders the parasite susceptible to normal human serum (136, 137). TgsGP inhibits APOL1 toxicity though a hydrophobic β-sheet (136) that initiates membrane stiffening, which could inhibit APOL1 membrane insertion leading to APOL1 degradation by endosomal proteases (128).

### Trypanosome-Suppressive Immunomodulating Factor (TSIF)

African trypanosomes (specifically *T. brucei*) encodes a suppressive protein, Trypanosome-suppressive immunomodulating factor (TSIF) that has been shown to have immunosuppressive actions *in vivo* (138). Treatment of mice with recombinant TSIF resulted in the activation macrophages that predominantly produce TNF-α and NO, suggesting that this protein favors the classical activation of macrophages (138). TSIF has also been shown to significantly impair Th2-induced inflammation in allergic asthma model, which further strengthens its ability to preferentially induce a Th1 inflammatory condition (139). Although, classical macrophage activation could be important for trypanosome clearance during the early phase of infection, a switch to a more alternatively-activated macrophage phenotype toward the late stage infection is required to prevent excessive inflammation and death of infected mice (33, 34, 37, 84). By employing the use of TSIF, trypanosomes are able to manipulate the host immune system by sustaining the classical macrophage activation during infection thereby suppressing the development of alternatively activated macrophages (Th2 condition). This could lead to exacerbated inflammation and death of infected animals.

## Concluding Remarks

**Figure 1:**
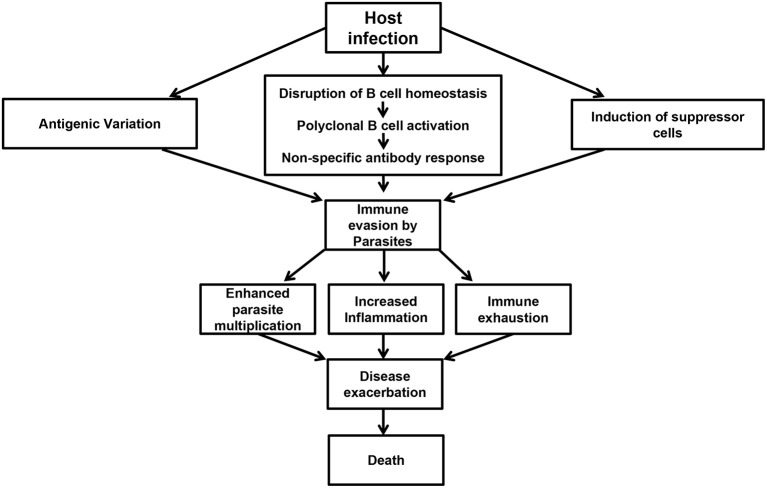
Events preceding immune evasion by parasites. Following infection, trypanosomes are able to undergo antigenic variation, disrupt B cell homeostasis, and initiate polyclonal B cell activation leading to the production of non-specific antibody responses. In addition, the infection is also associated with the induction of suppressor cells like Tregs and MDSCs. These events contribute to immune evasion by the parasites resulting in poor parasite control, increased inflammation, immune exhaustion, disease exacerbation, and death in untreated animals.

There is currently no vaccine against African trypanosomiasis and the current drugs for treatment of the disease are relatively ineffective. In addition to being toxic and expensive, the effectiveness of the current drugs is further hampered by increased risk of drug resistance and disease relapse (140–142). The development of a vaccine against this disease has been made virtually impossible due to antigenic variation and the difficulty in understanding the factors that regulate this and other important and highly complex host immune evasion mechanisms (38). Given that decades of research have failed to develop a vaccine against African trypanosomiasis, it is imperative that current research strategies have not yielded meaningful information regarding the induction and maintenance of effective and/or protective immune response to these parasites (143). Therefore, a change in strategy and our current way of thinking (including experimental designs and animal models) is needed. Studies focusing on halting various events that contribute to impaired immune response and immune evasion strategies by the parasites (Figure 1) could represent a major pathway toward disease control.

Because using the intraperitoneal route of infection does not mimic or represent the activities that take place during natural infection (which is initiated via intradermal inoculation by the tsetse fly), a switch to a more natural intradermal route of infection could be more beneficial in understanding key immune responses to these parasites, and could provide helpful information regarding vaccine development (25, 144). Furthermore, the causes of polyclonal B cell activation need to be further investigated since the parasite's ability to induce non-specific B cell responses to enhance their survival in the host still poses a great challenge in vaccine discovery. Although data showing the importance of optimal germinal center reaction during infection are beginning to appear (65), similar studies aimed at understanding other factors that regulate the parasite's ability to induce non-specific antibody responses could be beneficial. We showed that the ability of CD4^+^ T cells to proliferate and produce IFN-γ during *T. congolense* infection was inhibited by myeloid-derived suppressor cells in an arginase-1-dependent manner (122). This identifies arginase-1 as a potential target in African trypanosomiasis when considering treatment and vaccination strategies (144). It would be highly interesting to determine whether these exciting and novel mechanisms of immune regulation during *T. congolense* infections operate during infection with other African trypanosome species, particularly those that cause disease in humans.

In addition to the ongoing search for anti-parasite vaccine, adopting the anti-disease vaccine approach (145) could also serve as a great option to explore in African trypanosomiasis. This is based on the fact that trypanosome-associated factors could be targeted and nullified, which in turn would limit pathologies that are the major cause of disease. In line with this, cysteine peptidases have been identified as a potential candidate for anti-disease vaccine target (146) as they have been proposed to be likely involved in inducing alterations and pathologies that result in immunosuppression, anemia, and CNS disorder (147–150). Also, because the VSG GPI anchor induces very strong proinflammatory cytokine response that contributes to disease and pathologies (151, 152), it has been suggested that the GPI could be a promising target for anti-disease vaccine development (153). Indeed, it has been shown that GPI-based treatment alleviates trypanosomiasis-associated immunopathology, including anemia, weight loss, liver damage, inflammation, and proinflammatory cytokine production resulting in prolonged survival (154, 155). Although the efficacy of using synthetic GPI as vaccine is yet to be evaluated in infections caused by African trypanosomes, a study conducted in a mouse model of severe malaria using chemically-synthesized *Plasmodium falciparum* GPI glycan for immunization led to a significant reduction in early death and malaria-associated pathologies during challenge infection (153). This was associated with *in vitro* antibody (against GPI)-dependent neutralization of *P. falciparum*-induced inflammation (153). It would be interesting to investigate the impact of vaccination with synthetic GPI in the pathogenesis of experimental African trypanosomiasis, including the production of disease-enhancing proinflammatory cytokines.

## Author Contributions

CO: literature search and drafting. JU: provided corrections to the draft.

### Conflict of Interest

The authors declare that the research was conducted in the absence of any commercial or financial relationships that could be construed as a potential conflict of interest.
